# One year survival of ART and conventional restorations in patients with disability

**DOI:** 10.1186/1472-6831-14-49

**Published:** 2014-05-07

**Authors:** Gustavo F Molina, Denise Faulks, Ignacio Mazzola, Jan Mulder, Jo E Frencken

**Affiliations:** 1 Cátedra de Materiales Dentales, Facultad de Odontología, Universidad Nacional de Córdoba, Av. Maipú 177 4, B – 5000 Córdoba, Argentina; 2 CHU Clermont-Ferrand, Service d’Odontologie and Clermont Université, Université d’Auvergne, EA4847 Clermont Ferrand, France; 3 Department of Global Oral Health, College of Dental Sciences, Radboud University Medical Centre, Nijmegen, The Netherlands

**Keywords:** Disability and oral health, Atraumatic restorative treatment, Dental care for disabled, Professional practice, Dental caries, Glass-ionomer cement

## Abstract

**Background:**

Providing restorative treatment for persons with disability may be challenging and has been related to the patient’s ability to cope with the anxiety engendered by treatment and to cooperate fully with the demands of the clinical situation. The aim of the present study was to assess the survival rate of ART restorations compared to conventional restorations in people with disability referred for special care dentistry.

**Methods:**

Three treatment protocols were distinguished: ART (hand instruments/high-viscosity glass-ionomer); conventional restorative treatment (rotary instrumentation/resin composite) in the clinic (CRT/clinic) and under general anaesthesia (CRT/GA). Patients were referred for restorative care to a special care centre and treated by one of two specialists. Patients and/or their caregivers were provided with written and verbal information regarding the proposed techniques, and selected the type of treatment they were to receive. Treatment was provided as selected but if this option proved clinically unfeasible one of the alternative techniques was subsequently proposed. Evaluation of restoration survival was performed by two independent trained and calibrated examiners using established ART restoration assessment codes at 6 months and 12 months. The Proportional Hazard model with frailty corrections was applied to calculate survival estimates over a one year period.

**Results:**

66 patients (13.6 ± 7.8 years) with 16 different medical disorders participated. CRT/clinic proved feasible for 5 patients (7.5%), the ART approach for 47 patients (71.2%), and 14 patients received CRT/GA (21.2%). In all, 298 dentine carious lesions were restored in primary and permanent teeth, 182 (ART), 21 (CRT/clinic) and 95 (CRT/GA). The 1-year survival rates and jackknife standard error of ART and CRT restorations were 97.8 ± 1.0% and 90.5 ± 3.2%, respectively (p = 0.01).

**Conclusions:**

These short-term results indicate that ART appears to be an effective treatment protocol for treating patients with disability restoratively, many of whom have difficulty coping with the conventional restorative treatment.

**Trial registration number:**

Netherlands Trial Registration: NTR 4400

## Background

A recent systematic review revealed an equal to lower prevalence of dental caries in adults with disability when compared to the general population [1]. The major differences for the group with disability were a higher number of untreated carious lesions, lack of oral care and infrequent use of preventive strategies [1,2]. In another recent study of adolescents and adults, the authors observed that patients with intellectual disability had more decayed and missing teeth, fewer restorations and a greater need for tooth extraction than their siblings [3].

Many environmental barriers exist to access to oral health care in the population with disability. Even if these barriers are overcome, and the patient is able to find a dentist willing and able to treat, challenges remain. The provision of high quality restorative treatment is related to the patient’s ability to cope with the anxiety engendered by treatment and to cooperate fully with the demands of the clinical situation. Between a quarter and a third of adults with intellectual disability are estimated to have dental anxiety [4-6]. Unpleasant stimuli, such as the injection of local anaesthesia or the noise and vibration of rotary instruments, may provoke disproportionate anxiety and subsequent opposition to treatment. In addition, poor muscle coordination, fatigability or oral dysfunction such as drooling and tongue movement, may compromise restorative procedures. Sedation or general anaesthesia may improve clinical conditions for restorative work but these techniques have their own problems in terms of cost and patient morbidity [7].

A less anxiety-provoking restorative treatment is Atraumatic Restorative Treatment (ART). This approach is endorsed by the World Health Organisation and involves hand instrumentation and placement of high-viscosity glass-ionomer cement restorations. ART has been shown to be equally effective as conventional restoration in both primary and permanent teeth [8]. It has been suggested that ART might help to reduce barriers to treatment for patients with disabilities [9,10] but no trial comparing ART with conventional treatment in this population has yet been reported.

The aim of the present study was to assess the survival rate of ART restorations compared to conventional restorations in patients with disability referred for special care dentistry. In addition, this report aims to outline the methods used to obtain and analyse the data in detail, as a basis for future reports.

## Methods

### Ethics

Ethical approval was obtained from the local Ethical Committee, CIEIS Facultad de Odontología, Universidad Nacional de Córdoba with the reference number 38/2012 and the trial was registered at the Netherlands Trial register with number NTR 4400.

### Participants

All patients with a disability referred for restorative treatment to the Dental Hospital of the National University of Córdoba, Argentina over a six month period were considered for inclusion in the study. Patients were examined by one of two special care dentists. Medical history was taken. A full description of the functional, social and environmental context of the patient was recorded using the International Classification of Functioning Oral Health Checklist [11].

Clinical examination included: 1) report of pain by the patient and/or caregiver, and targeted examination of potentially painful teeth; 2) presence of dental plaque, assessed according to the criteria of Greene and Vermillion [12] and recorded using the Simplified Oral Hygiene Index (S-OHI); 3) gingival bleeding, measured on buccal and lingual surfaces of all teeth according to the criteria of Ainamo and Bay [13] and recorded using the gingival bleeding index (GBI) and; 4) dental caries according to the criteria of the World Health Organization (WHO) recorded as mean dmft/DMFT scores [14].

### Development of information brochures

Two brochures were prepared, one explaining the conventional restorative treatment (CRT) and one the Atraumatic Restorative Treatment (ART) protocols. The brochures were prepared by the research team and consisted of a brief description of each approach, the steps followed for each procedure, their advantages and disadvantages using essential information obtained from current text books of Operative Dentistry and the literature [15,16], explained in lay terms. Pictures and figures were selected from a pool of images provided by the members of the team. A first lay-out of the brochure was discussed and modified by a group of five experts in special care dentistry during two focus group discussions at the Paediatric Department, Dental School, Catholic University of Córdoba. Thereafter, the appropriateness of the brochures was piloted with 30 patients, attending six special care clinics in Córdoba, whose specialists did not participate in the focus group. Feedback led to a preliminary version of the brochure which underwent content validation at the annual meeting of the Argentinean Association of Disability and Oral Health. Thirty-four delegates, representing different counties, were sent information regarding the aims of the clinical study and the preliminary brochures, one week prior to the meeting to prepare their suggestions. Discussion was held in a special session of the meeting, where participants made some suggestions for improving the content and lay-out of the brochures. Finally, consensus was reached for minor adjustments, and unanimous approval was given regarding both content and lay-out of the two brochures.

### Study design and attribution to treatment group

The following inclusion criteria applied: patient with a recognised disability and at least one dentine carious lesion in a primary or permanent tooth without pulpal involvement and without spontaneous pain or tooth mobility, but in occlusion with the antagonist tooth or teeth and in contact with the neighbouring tooth or teeth.

Randomisation of persons with intellectual disability in clinical trials raises legitimate ethical concerns relating to ability to fully inform and the value of proxy consent [17,18]. In order to avoid this problem, the treatment selection process was conducted as follows: The study aims and design were explained to the patients and/or the parents or caregivers (hitherto referred to as ‘respondents’) of all those eligible for inclusion. Individual treatment needs were explained verbally by the dentist. Standardised verbal information and the two validated brochures were used to present the respondents with the treatment options, and the dentists were instructed to be as neutral as possible during this presentation. Respondents kept the brochures to read at home and it was unlikely that they would have the possibility to exchange views.

At the second visit, respondents confirmed their choice of either ART or CRT and provided written informed consent for participation in the study. Researchers recorded the reasons that led them to choose either one or the other option, in order to identify their expectations and perceived barriers regarding a dental procedure.

### Treatment procedures

*Conventional restorative treatment (CRT):* Dentine carious lesions in primary and permanent teeth were restored after infiltration of local anaesthesia using rotary instruments with high-speed carbide burs (#330 and #245, KG Sorensen, Cotia-SP, Brazil) under rubber dam isolation. Remaining carious tissues were removed using low-speed round burs (#1, #2, #3, KG Sorensen, Cotia-SP, Brazil). Calcium hydroxide cement was applied on the floor of deep cavities only. Proximal cavities were contoured with a metal band and wooden wedges. Cavities were prepared using an adhesive system (Scotchbond Multipurpose, 3 M ESPE, St. Paul, Minnesota, USA), light-cured for 20 sec and restored incrementally with composite resin (Filtek Z250, 3 M ESPE, St. Paul, Minnesota, USA) in layers of less than 2 mm, and light-cured for 40 sec with a LED lamp (Elipar™ FreeLight 2 LED Curing Light, 3 M ESPE, St. Paul, Minnesota, USA). Occlusal anatomy was carved with hand instruments before light-curing. Restoration adjustment was performed with diamond finishing burs (KG Sorensen, Cotia-SP, Brazil) and polished with rubber tips and fine disks.

*Atraumatic Restorative Treatment (ART*): Soft demineralised carious tissues were removed from dentinal lesions in primary and permanent teeth using hand instruments only (ART Kit; Henry Schein, Chicago, USA) according to the ART protocol [14]. In proximal cavities, a steel matrix band (Palodent, Denstply Caulk, Milford, DE) and wooden wedges were used. 10% polyacrylic acid (dentine conditioner, GC America, Chicago, USA) and wet and dry cotton wool pellets were used to condition and dry the cavity. Under cotton roll isolation, cavities were restored with one of the two encapsulated high-viscosity glass-ionomer cements: EQUIA system (GC, Tokyo, Japan) or Chemfil Rock (Dentsply/De Trey, Konstanz, Germany). The type of cement used was randomised between patients as follows: A flip of a coin determined which cement was used in the first patient. The other material was then applied in the second patient, and this sequence was followed until the last patient had been treated. Capsules were activated according to manufacturers´ instructions. The cavity and adjacent fissures were filled and held under finger pressure for 60 sec. Excess cement was removed with hand instruments. G-Coat (GC, Tokyo, Japan) was applied over EQUIA cement and cured for 10 sec and a layer of petroleum jelly was placed over the Chemfil Rock restorations for maintaining the water balance in the glass-ionomer cement during setting.

*Conventional restorative treatment under general anaesthesia (CRT/GA)*: Restorative treatment was the same as described under CRT. Local anaesthesia was administered only when tooth extractions were indicated.

### Provision of treatment

At the second visit, the operator performed the selected treatment. This led to the following situations: 1) The patient was able to cope with the dental treatment and the operator was able to place the restorations with the chosen treatment to an acceptable clinical standard. If further restorations were needed, additional sessions were scheduled using the same treatment; 2) The patient was unable to cope with the dental treatment and the operator was, therefore, unable to place the restorations with the chosen treatment to an acceptable clinical standard. If further restorations were needed, treatment was programmed using the alternative treatment; 3) The patient was unable to cope with either treatment approach and the patient was referred for conventional treatment under general anaesthesia (GA).

### Evaluation

The quality of the restorations was assessed by two calibrated independent examiners at 6 and 12 months using established ART restoration criteria (codes 0–6) [16] with the addition of one code for determining ‘pulpal involvement’. A lesion was scored carious if it had penetrated the dentine. For the calibration process, ten patients presenting 48 restorations were double-blind assessed independently by the two examiners. The inter-examiner consistency, expressed as kappa coefficient and the percentage of agreement (P_o_), was 0.62 (CI:0.30-0.95) and 91.7%, respectively.

### Statistical analysis

Data were entered into a data base and analysed using SAS 9.2 software by a statistician from the College of Dental Sciences in Nijmegen, the Netherlands. Restorations with codes 0 and 1 (sound, and small defect at the restoration margin) were considered to have survived. All other codes were considered failures. Presence of a dentine carious cavity alongside the restoration (secondary caries) was considered a failure. The dependent variable was the survival of restorations. Independent variables were: treatment group (ART; CRT/clinic; CRT/GA); type of teeth (primary; permanent); type of surface (single-; multiple-surface); gender; age; operator (1;2); glass-ionomer (Chemfil Rock; EQUIA system); number of primary and permanent teeth restored per person; mean dmft-, mean dt-score, mean DMFT- and mean DT-score at baseline; mean plaque score; and gingival bleeding score. ANOVA and chi-square tests were used to test for differences between independent variables at baseline. The Proportional Hazard Rate Regression Model [19] with frailty correction [20] was used to estimate cumulative survival rates of ART and CRT restorations. The Wald test (chi-square) was used to test for differences in survival rates. The Jackknife method [21] was applied to calculate standard errors. Statistical significance was set at α = 0.05.

## Results

### Disposition of subjects

A total of 66 patients were included in the study, 36 male (54.5%) and 30 female (45.5%), with a mean age of 13.6 (±7.8) years, ranging from 3- to 39-years old. There were 16 different principal medical diagnoses. The most common principal medical diagnosis was Cerebral Palsy (39.4%), followed by Autistic Spectrum Disorder (19.7%), West syndrome (9.1%), Down syndrome (6.1%), Mental Retardation of unspecified origin (6.1%) and Rett syndrome (4.5%). Ten patients had different, less frequently occurring medical disorders (15.2%). Cerebral palsy was the most common disorder amongst patients treated with ART (51.1%) followed by the infrequently occurring disorders (17.0%) and Autistic Spectrum Disorder (12.8%). The latter was the most common disorder amongst those patients treated under GA (42.9%).

Mean DMFT and dmft-score were 17.3 ± 11.9 and 7.8 ± 8.6, respectively, whilst the prevalence of plaque and gingival bleeding in this population with disability was 100%. Fifty-two percent of the total population had a mean plaque score of at least 1.5 and 48.0% had at least 35% of their teeth affected by gingival bleeding.

**Figure 1:**
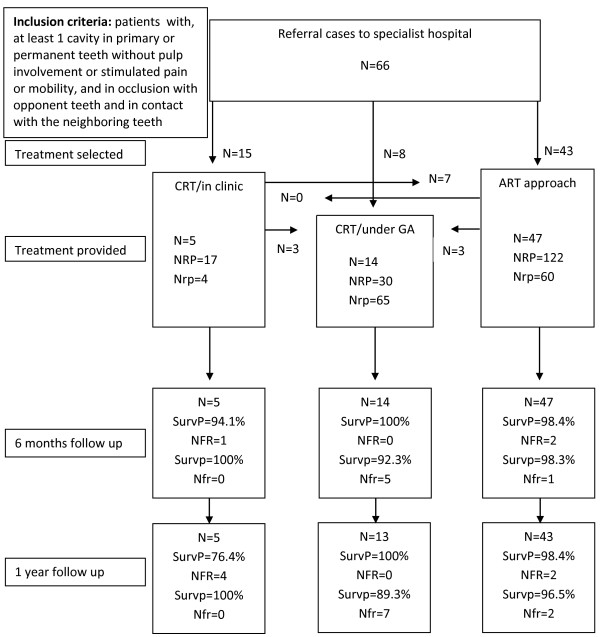
Flow chart of patients, number of restorations, restoration survival rates by treatment group and evaluation of the study group.

One operator treated 35 and the other 31 patients. The total number of restorations placed was 298: 105 (ART: Chemfil Rock), 77 (ART: EQUIA system), 21 (CRT/clinic) and 95 (CRT/GA). ART treatment was selected by 43 respondents and 15 respondents chose conventional treatment in the clinic. Treatment in the clinic was deemed unfeasible from the outset for 8 patients (as full initial examination was impossible) and these patients were referred to GA for conventional treatment. Five patients, with 15 restorations, dropped out at year one. The flow chart of patients, number of restorations, restoration survival rates by treatment group and evaluation period is presented in Figure 1*.*

### Effect of background variables on the treatment groups

*ART treatment group:* Gender distribution was 55.0% (males) and 45.0% (females). One operator treated 53.0% and the other 47.0% of the patients receiving ART. A total of 60 ART restorations (46 Chemfil Rock and 14 EQUIA system) were placed in primary teeth and 122 ART restorations (59 Chemfil Rock and 63 EQUIA system) in permanent teeth. Local anaesthesia was provided for 9.0% of patients. There were no statistically significant differences observed between the two groups of ART restorations for the independent variables at baseline, except for gingival bleeding (p = 0.02).

*CRT treatment groups:* Gender distribution was 53.0% (males) and 47.0% (females). One operator treated 53.0% and the other 47.0% of the patients receiving CRT. A total of 69 restorations (4 in CRT/clinic and 65 in CRT/GA) were placed in primary teeth and 47 restorations (17 in CRT/clinic and 30 in CRT/GA) were placed in permanent teeth. Local anaesthesia was provided for 89.0% of the patients treated with the CRT protocol. There were no statistically significant differences between the two groups of CRT restorations at baseline for age (p = 0.14), number of primary (p = 0.39) and permanent teeth (p = 0.07) restored per person, mean dmft-score (p = 0.10), mean DMFT- (p = 0.14) and mean DT-score (p = 0.07), mean plaque score (p = 0.86) and for gingival bleeding effect (p = 0.86). There was a mean dt-score effect (p = 0.04). Except for this latter variable, the analysis showed no statistically significant difference in background variables between the two CRT groups.

The effect of background variables, their mean and standard deviations at baseline for the ART, CRT/clinic and CRT/GA groups is presented in Table 1. There were on average significantly more restorations in permanent teeth placed in patients treated under GA than those treated using the other treatment protocols (p = 0.001). The patients receiving GA also had a significantly higher percentage of teeth with bleeding gums than those in the other groups (p = 0.02).

**Table 1:** Effect of background variables at baseline according to the three treatment groups

**Background variables at baseline**	**ART**	**CRT**		**p-value**
	**Clinic**	**Clinic**	**GA**	
Mean age	13.7 ± 8.1	17.6 ± 7.6	11.8 ± 7.0	0.36
Number of patients	47	5	14	
Number of males	26	0	10	
Mean dmft ± SD	7.3 ± 8.0	4.0 ± 8.9	12.0 ± 8.8	0.10
Mean dt ± SD	1.9 ± 3.0	0.4 ± 0.9	5.2 ± 4.7	0.07
Mean ft ± SD	0.2 ± 0.9	0.0 ± 0.0	0.2 ± 0.9	0.87
Mean DMFT ± SD	17.9 ± 10.7	22.0 ± 12.8	11.9 ± 12.4	0.13
Mean DT ± SD	3.0 ± 3.0	7.2 ± 6.4	2.7 ± 3.7	0.78
Mean FT ± SD	0.4 ± 1.4	1.4 ± 1.9	0.4 ± 1.6	0.38
Mean plaque score ± SD	1.7 ± 0.5	1.9 ± 0.7	2.0 ± 0.7	0.27
Gingival bleeding (%) ± SD	35.3 ± 10.3	40.0 ± 15.8	46.1 ± 16.2	0.02*

N = number; SD = Standard Deviation; ART = Atraumatic Restorative Treatment; CRT = Conventional Restorative Treatment; GA = General anaesthesia; prim teeth = primary teeth; perm teeth = permanent teeth; dmft = decayed, missing and filled primary teeth; DMFT = decayed, missing and filled permanent teeth.

*Statistically significant difference.

### Survival of restorations by ART groups

The 1-year survival rates for ART/Chemfil Rock and ART/EQUIA system restorations in primary teeth were 95.4% and 100%, respectively, whilst the 1-year survival rates for restorations in the permanent teeth were 98.4% (ART/Chemfil Rock) and 98.3% (ART/EQUIA system). There were no significant differences in survival between the two groups of ART restorations, other than for the EQUIA system group, which is inevitable as 2 failed restorations are worse than no failures. The two groups were therefore combined for comparison with the CRT restorations.

### Survival of restorations by CRT groups

The 1-year survival rates of CRT/clinic and CRT/GA restorations in primary teeth were 100% and 89.3%, respectively, whilst the 1-year survival rates of CRT/clinic and CRT/GA restorations in permanent teeth were 76.4% (CRT/clinic) and 100% (CRT/GA). The differences in survival rates between the two treatment groups for both types of teeth over the one year period were statistically significant (p < 0.0001).

### Comparison of ART and CRT restorations

A six month follow up was appointed to identify early failures or emergency cases. During that period, one permanent tooth belonging to the ART/Chemfil Rock group had to be extracted due to an acute infection. Seven restorations of the CRT/in clinic group identified as early failures (codes 2 and 3) as well as one restoration of the ART/EQUIA group that had been lost were therefore replaced. At one year, the survival rates and jackknife standard errors of all ART and CRT restorations were statistically significantly different: 97.8 ± 1.0% and 90.5 ± 3.2% (p = 0.01), respectively. Table 2 shows the survival rates and jackknife standard errors of ART and CRT restorations by type of teeth. Corrected for a possible effect of type of surface, the Wald test did not show a statistically significantly difference between ART and CRT restorations placed in primary (p = 0.29) and in permanent teeth (p = 0.19) over the one year survival period. The survival rates and jackknife standard errors of single- and multiple-surfaces ART and CRT restorations in primary and in permanent teeth over the one year period are presented in Table 3.

**Table 2:** Percentage survival rates (surv) and jackknife standard error (SE) of ART and CRT restorations by type of teeth

**Interval (years)**	**ART**				**CRT**			
	**primary**		**Permanent**		**primary**		**Permanent**	
	**surv**	**SE**	**surv**	**SE**	**surv**	**SE**	**surv**	**SE**
0.5	98.3	0.6	98.4	1.2	92.8	5.1	97.8	0.7
1.0	96.5	2.6	98.4	1.2	89.9	4.1	91.3	7.2

ART = Atraumatic Restorative Treatment; CRT = Conventional Restorative Treatment.

**Table 3:** The survival rates (surv) and jackknife standard errors (SE) of single- and multiple-surface ART and CRT restorations in primary and in permanent teeth over the one year period

**Interval (years)**	**ART**				**CRT**			
	**Single**		**multiple**		**single**		**Multiple**	
	**surv**	**SE**	**surv**	**SE**	**surv**	**SE**	**surv**	**SE**
Primary teeth								
0.5	100	0	92.6	4.0	94.7	1.6	90.0	7.4
1.0	100^a^	0	84.3	1.2	94.7^b^	1.6	83.4	6.5
Permanent teeth								
0.5	100	0	92.3	5.7	100	0	93.2	2.7
1.0	100	0	92.3^c^	5.7	100	0	71.8^d^	21.5

P^a,b^ = 0.03; P^c,d^ = 0.30.

### Reasons for failure

Five restorations failed because of a marginal defect of > 0.5 mm (code 2), 6 failed because of a fracture in the restoration (code 3), 2 failed because the restoration was absent, 1 because other treatment had been performed (code 5) and 1 failed because an abscess had developed. Two single-surface CRT restorations failed in anterior primary teeth, 3 multiple-surfaces CRT restorations failed in posterior primary teeth, 2 multiple-surfaces CRT restorations failed in anterior primary teeth and 2 multiple-surfaces ART restorations failed in posterior primary teeth. Of the multiple-surface CRT restorations in permanent teeth, 3 failed in anterior and one in a posterior tooth. One multiple-surfaces ART restoration failed in an anterior permanent tooth.

## Discussion

The current study reports a significantly higher survival rate for all ART restorations compared to all CRT restorations over the one year period. This finding confirms previous reports of longevity of ART restorations in children and adolescents in different clinical settings [8], and supports WHO endorsement of the approach. Although long term follow up is required, cumulative survival rates for single and multiple-surfaces ART restorations obtained in this clinical study were higher than the results of a meta-analysis for ART restorations [22] and consistent with a controlled clinical trial in primary molars at similar time intervals [23]. The use of enhanced glass-ionomer cements is likely to be responsible for such an improvement. It is true that the use of restorative high-viscosity glass-ionomer cements might be a matter of concern when using the ART approach, especially in stress bearing situations. Biomimetic features of this material are usually undermined by their poor mechanical properties. Therefore, several in vitro studies were performed before starting this clinical trial which concluded in the specific selection of the two encapsulated restorative high-viscosity glass-ionomer cements used here [24,25].

In terms of the use of high-viscosity glass-ionomer cements specifically in the population with special needs, the current study joins the encouraging results reported by Gryst and Mount [26] who used restorative high-viscosity glass-ionomer cements (conventional and resin modified) in 174 patients with intellectual and/or physical disability. Clinical procedures were not standardised in this study, however, so results are difficult to generalise. ART was mentioned as a potential strategy by these authors and was later tested by Molina and Kultje [27], assessing the influence of a chemo-mechanical caries removal system to enhance clinical performance of ART restorations in patients with intellectual disability over 1 year. The outcome of this study stressed the importance of optimal caries removal to achieve long term survival of restorations, although the critical influence of the restorative material is also recognised [8].

Of the 15 restorations out of 298 that failed in the current study, failure was most often related to a marginal defect and fracture in the restoration. Moisture control may be particularly problematic during restorative treatment for persons with disability. Hypersalivation, dysfunctional swallowing, tongue movement, inability to keep still over short periods and difficulty accepting rubber dam for CRT may all result in contamination of a prepared cavity by saliva. In addition, poor periodontal health and gingival bleeding may also cause technical problems on placement of restorative materials. The prevalence of gingivitis amongst the patients with disability has been reported at almost 100%, and 48.0% of patients in the current study had a GBI of over 35.0%. Both saliva and blood will reduce the adhesive properties of the restorative material used, whether composite resin or glass-ionomer cement. It would be assumed that this challenge was overcome during placement of restorations under GA, but the results only showed the absence of a significant difference in the survival of restorations in permanent but not in primary teeth placed under general anaesthesia. Failure was shown to be related to the extent of the lesion to be restored, however, with larger restorations faring worse than their smaller counterparts. This too, confirms previous results involving large study populations whether for ART [21] or conventional treatment outcomes [28]. There was also a remarkable difference in the need to administer local anaesthesia between people treated with hand instruments (ART) and rotary instrumentation (CRT). The reduced need for local anaesthesia with ART is in line with results obtained from other studies in which ART was compared to CRT in children [29] and adolescents [30].

The current study is one of very few clinical trials ever to compare different restorative treatment outcomes in special care dental patients. Special care dentistry is gradually gaining recognition as a specialty in its own right around the world, but the evidence base is still lacking for the adaptation of certain clinical techniques to the needs of this population. Clinical research involving the population with disability is notoriously difficult, mainly in relation to legitimate concerns over informed consent. These problems may be compounded when investigating alternative therapeutic approaches that may be perceived as ‘second-class’ treatment options. This point of view was expressed in a previous survey of special care dentists’ attitudes to ART, with 30.0% of respondents perceiving ART as ‘lower quality dentistry’ [10]. Five respondents went as far as to say that they would probably not use ART even if ‘reliable scientific evidence showed the suitability and effectiveness of the ART approach in this population’. The results of the current trial refute this persistent image of the ART approach as substandard treatment.

In order to avoid some of the problems discussed above, the current study was not designed as a randomised control trial, but respondents were encouraged to choose the approach they felt was most appropriate for the individual patient. The authors acknowledge that this may weaken the study methodology, but if original treatment strategies are required for this unique group of patients, then so are original research strategies! The fact that 73.0% of respondents chose the ART approach confirms the attractiveness of a technique that avoids the drill, the principal advantage of ART perceived by special care dentists in the survey by Molina et al. [10]. It is also probable that a certain number of the patients, who successfully received ART restorations here, would have required GA for placement of conventional restorations. The current study was not designed to test this hypothesis, but it would be an interesting theory to test in subsequent trials as it raises inevitable questions relating to the cost and morbidity associated with providing restorative treatment under GA.

In addition to the non-random assignment of patients to restorative strategies, the study presents a certain number of other limitations. No power calculation could be performed resulting in an unevenly distributed sample size over the treatment group, which was small in terms of numbers of patients despite a large number of restorations performed. In addition, no common randomisation of patients to a treatment group could be performed. However, the attribution of a person to a treatment group was undertaken as arbitrarily as possible, by eliminating the influence of the dentist in this decision making process as far as possible. Furthermore, the operators could obviously not be blinded to the treatment group [31]. Although these are important considerations, it must be emphasised that the survival of the restorations was assessed by two independent observers, that the inter-consistency test was substantial [32] and that the survival analyses took into account the dependency of repeat restorations for a single patient. Therefore, the findings of the present study are valid to a high standard considering the nature of the study population.

## Conclusion

The present study showed that ART restorations using Chemfil Rock and EQUIA system survived longer than composite resin restorations over a one year period in patients with disability. It is hoped that this evidence may help special care dentists to overcome their mistrust of the ART approach and encourage them to add this treatment concept to their therapeutic arsenal. The ART approach has the potential not only to improve patient experience of dental treatment, but also to reduce health costs and patient morbidity by reducing referrals for GA. It is now essential to build a stronger evidence base to confirm or refute the potential benefits of the ART approach for persons with disability suggested by the current results.

## Competing interests

The authors declare no conflict of interests for the report of the results obtained in the present study. Disclaimer: The views expressed in the submitted article belong to the authors and are not an official position of the institution or funder.

## Authors’ contribution

GFM and JEF designed the study, IM and GFM implemented the study, JM, GFM and JEF analysed the data, GFM, DF, JEF wrote the manuscript. All authors approved the final version of the manuscript.

## Pre-publication history

The pre-publication history for this paper can be accessed here:

http://www.biomedcentral.com/1472-6831/14/49/prepub
